# Regional distribution of serological evidence of vaccine-induced immunity to hepatitis B among cancer patients in a city in southeastern Brazil

**DOI:** 10.1590/S1678-9946202668066

**Published:** 2026-10-02

**Authors:** Victória Gonçalves de Paula, Marina Rossi de Camargo Pinto, Gustavo Manoel Ferreira, Débora Bignotto Rosane Battaglia, Rafael de Oliveira, Caroline Manchiero, Bianca Peixoto Dantas, Fátima Mitiko Tengan, Edson Abdala, Mariana Cavalheiro Magri

**Affiliations:** 1 Universidade de São Paulo, Faculdade de Medicina, Hospital das Clínicas, Laboratório de Investigação Médica em Hepatologia por Vírus (LIM-47), São Paulo, São Paulo, Brazil; 2 Universidade de São Paulo, Faculdade de Medicina, Instituto do Câncer do Estado de São Paulo, São Paulo, São Paulo, Brazil; 3 Universidade de São Paulo, Faculdade de Medicina, Instituto de Medicina Tropical de São Paulo, São Paulo, São Paulo, Brazil; 4 Universidade de São Paulo, Faculdade de Medicina, Departamento de Infectologia e Medicina Tropical, São Paulo, São Paulo, Brazil

**Keywords:** Hepatitis B antibodies, Epidemiology, Neoplasms, Urban area.

## Abstract

Vaccination is the primary strategy for preventing hepatitis B virus (HBV) infection, but its frequency remains insufficiently characterized among cancer patients. This study evaluated the regional distribution of serological evidence compatible with vaccine-induced immunity to hepatitis B among cancer patients residing in Sao Paulo city, Brazil. We conducted a retrospective observational study of adult patients who underwent hepatitis B surface antibody (anti-HBs) serological testing at the Instituto do Cancer do Estado de Sao Paulo (ICESP) in 2023. Serological evidence compatible with vaccine-induced immunity was defined as reactive anti-HBs and non-reactive hepatitis B core antibody (anti-HBc). Univariable and multivariable Poisson regression analyses were performed to identify associated factors. Among 1,143 patients, the mean age was 57.4 ± 16.42 years, 61.9% were female, 60.6% self-identified White, and 76.8% had solid tumors. Overall, 26.2% of cancer patients had serological evidence compatible with vaccine-induced immunity to hepatitis B. Descriptively, the North Zone had a lower frequency than the South Zone (20.7% versus 28.9%), although regional differences were not statistically significant after multivariable adjustment. Female sex (adjusted prevalence ratio [aPR] = 1.67, 95% CI: 1.23-2.27, p = 0.001) and younger age (18-39 years versus ≥ 60 years: aPR = 6.89, 95% CI: 4.72-10.95, p < 0.001) were independently associated with serological evidence compatible with vaccine-induced immunity. These findings indicate a low frequency of serological evidence compatible with vaccine-induced immunity to hepatitis B among cancer patients across Sao Paulo, particularly among older adults and men.

## INTRODUCTION

Globally, an estimated 0.9 million individuals were newly infected with hepatitis B virus (HBV) in 2024. From 2000 to 2024, Brazil reported 302,351 newly diagnosed cases of hepatitis B, of which 63,670 occurred in Sao Paulo State^1^. Sao Paulo city (23°32’51” S 46°38’10” W) has approximately 11.4 million inhabitants, distributed across five major urban zones: Central, East, North, West, and South. Concurrently, Brazil recorded an estimated 627,193 new cancer cases in 2022^2^. Within this context, preventive strategies are critical for patients with cancer, who are at increased risk of HBV reactivation and other infection-related complications driven by disease- and treatment-related immunosuppression^3^. In particular, anticancer therapies may predispose patients with previous HBV infection to viral reactivation and severe hepatic complications^3^.

Vaccination against HBV is the primary strategy for preventing HBV infection in both the general population and patients receiving anticancer therapy. In the general population, seroprotection rates after completion of the full vaccination schedule are expected to reach 98%-100% in children and 90%-95% in adults. However, vaccine-induced immune responses are less robust among individuals older than 40-50 years^4^. The isolated presence of hepatitis B surface antibody (anti-HBs), in the absence of anti-HBc, is widely accepted as serological evidence compatible with vaccine-induced immunity to hepatitis B. Therefore, this study aims to determine the frequency and regional distribution of serological evidence compatible with vaccine-induced immunity to hepatitis B among cancer patients residing in Sao Paulo city.

## MATERIALS AND METHODS

### Study design

This retrospective observational study included adult cancer patients receiving care at the Instituto do Cancer do Estado de Sao Paulo (ICESP), affiliated with the Clinical Hospital of the Faculty of Medicine, University of Sao Paulo (HCFMUSP), between January and December 2023. Serological evidence compatible with vaccine-induced immunity to hepatitis B, defined as immunity resulting from prior hepatitis B vaccination rather than previous infection, was considered present when anti-HBs was reactive and hepatitis B core antibody (anti-HBc) was non-reactive. All serological tests were performed using a chemiluminescent microparticle immunoassay (CMIA) on the Alinity i system (Abbott Diagnostics, Chicago, IL, USA) at the Central Laboratory Division of HCFMUSP. According to the laboratory’s routine procedures, an anti-HBs titer ≥ 10 IU/L was classified as reactive. Testing was conducted as part of routine clinical care before the study began, with data subsequently retrieved from the hospital’s medical records.

### Ethics

This study was approved by the Ethics Committee for Analysis of Research Projects of the HCFMUSP under protocols Nº 6.120.828 and 6.718.762 (CAAE: 70101521.9.0000.0068). Requirement for informed consent was waived, and all data were de-identified before analysis to ensure patient confidentiality.

### Data source

Patients with solid tumors or hematologic malignancies who attended the ICESP, aged 18 years or older and who underwent anti-HBs serological testing were eligible for inclusion. Patients without anti-HBc testing or with reactive anti-HBc or hepatitis B surface antigen (HBsAg) results were excluded, as were those who did not reside in Sao Paulo city. All eligible patients who met the inclusion and exclusion criteria during the study period (January to December 2023) were consecutively identified and included; therefore, no sampling strategy was applied.

Demographic characteristics (i.e., age, sex [sex assigned at birth], and ethnicity/skin color), primary cancer type (i.e., solid tumors or hematological malignancies), and residential zone within Sao Paulo city were retrieved from the hospital’s medical records. Ethnicity/skin color was classified according to participants’ self-identification using the Brazilian census classification system: Brazilians of African origin who self-identified as “Black,” Indigenous Brazilians who self-identified as “Indigenous,” Brazilians of mixed ethnic background who self-identified as “Pardo” (“Brown/Mixed-ethnicity”), Brazilians of European origin who self-identified as “White,” and Brazilians of Asian origin who self-identified as “Asian.”

### Statistical analysis

Age was analyzed both as a continuous variable and as a categorical variable (18-39, 40-59, and ≥ 60 years). Sex was classified as male or female; ethnicity/skin color as White or non-White (including Black, Indigenous, Pardo, and Asian); and primary cancer type as hematological malignancies or solid tumors.

Associations between zone of residence (Central, East, North, West, and South), epidemiological characteristics, and serological evidence compatible with vaccine-induced immunity to hepatitis B (anti-HBs) were evaluated by using univariable Poisson regression to estimate unadjusted prevalence ratios (PRs) and 95% confidence intervals (CIs). Consistent with established variable-selection procedures, variables with p-values < 0.20 in the univariable analyses were selected for inclusion in the multivariable Poisson regression model. Differences in demographic characteristics and cancer type across the five zones were assessed using Pearson’s chi-square test. Statistical analyses were performed using IBM SPSS Statistics for Windows, version 22.0 (IBM Corp., Armonk, NY, USA), and statistical significance was set at p < 0.05.

## RESULTS

In 2023, 2,792 adult cancer patients underwent anti-HBs serological testing at ICESP. Of these, 327 were excluded because anti-HBc testing had not been performed, 253 because they were anti-HBc-reactive, and 33 because they were HBsAg-reactive. Although 2,179 patients met the initial serological criteria, 1,036 were excluded because they resided outside Sao Paulo city. Consequently, the final study population comprised 1,143 patients who met all inclusion and exclusion criteria.

Among included patients, the mean age was 57.4 ± 16.42 years, 61.9% were female, and 60.6% self-identified as White. Solid tumors accounted for 76.8% of cases, whereas hematological malignancies accounted for 19.4%. Patients were distributed across all zones of the city, with the largest proportions residing in the South (29.7%) and East (25.0%) (Supplementary Table S1).

Overall, 26.2% (299/1,143) of cancer patients residing in Sao Paulo city showed serological evidence compatible with vaccine-induced immunity to hepatitis B. Descriptively, the highest proportions were observed in the South (28.9%, 98/339) and East (28.7%, 82/286), whereas the lowest was observed in the North (20.7%, 51/246). The regional distribution and corresponding 95% CI are presented in Figure 1.

In the univariable Poisson regression, serological evidence compatible with vaccine-induced immunity to hepatitis B was less frequently observed among patients residing in the North than those residing in the South (20.7% versus 28.9%; PR = 0.72, 95% CI: 0.51-1.01, p = 0.054).

However, this association was no longer statistically significant after adjustment for potential confounders in the multivariable Poisson model (p = 0.139), indicating no independent association between zone of residence and serological evidence compatible with vaccine-induced immunity. In contrast, age group and sex remained independently associated with the outcome (p < 0.05) (Table 1). Compared with patients aged ≥ 60 years, those aged 18-39 years were more likely to present serological evidence compatible with vaccine-induced immunity (adjusted PR [aPR] = 3.56, 95% CI: 2.66-4.77), whereas patients aged 40-59 years were also more likely to present the outcome (aPR = 1.73, 95% CI: 1.30-2.31). Likewise, female patients were more likely than male patients to present serological evidence compatible with vaccine-induced immunity (aPR = 1.42, 95% CI: 1.10-1.82).

No statistically significant differences were observed in the distribution of age (p = 0.184), sex (p = 0.504), ethnicity/skin color (p = 0.835), or primary cancer type (p = 0.340) across the five zones of Sao Paulo city (Table 2). Descriptively, the West had the highest proportion of younger adults (18-39 years, 20.5%), whereas the North had the highest proportion of older adults (≥ 60 years, 55.3%). Female patients, White patients, and patients with solid tumors predominated across all regions. Likewise, despite slight descriptive variation in the proportion of patients with serological evidence compatible with vaccine-induced immunity to hepatitis B across the five zones (Figure 1), no statistically significant regional differences were observed (p = 0.180).

**Figure 1:**
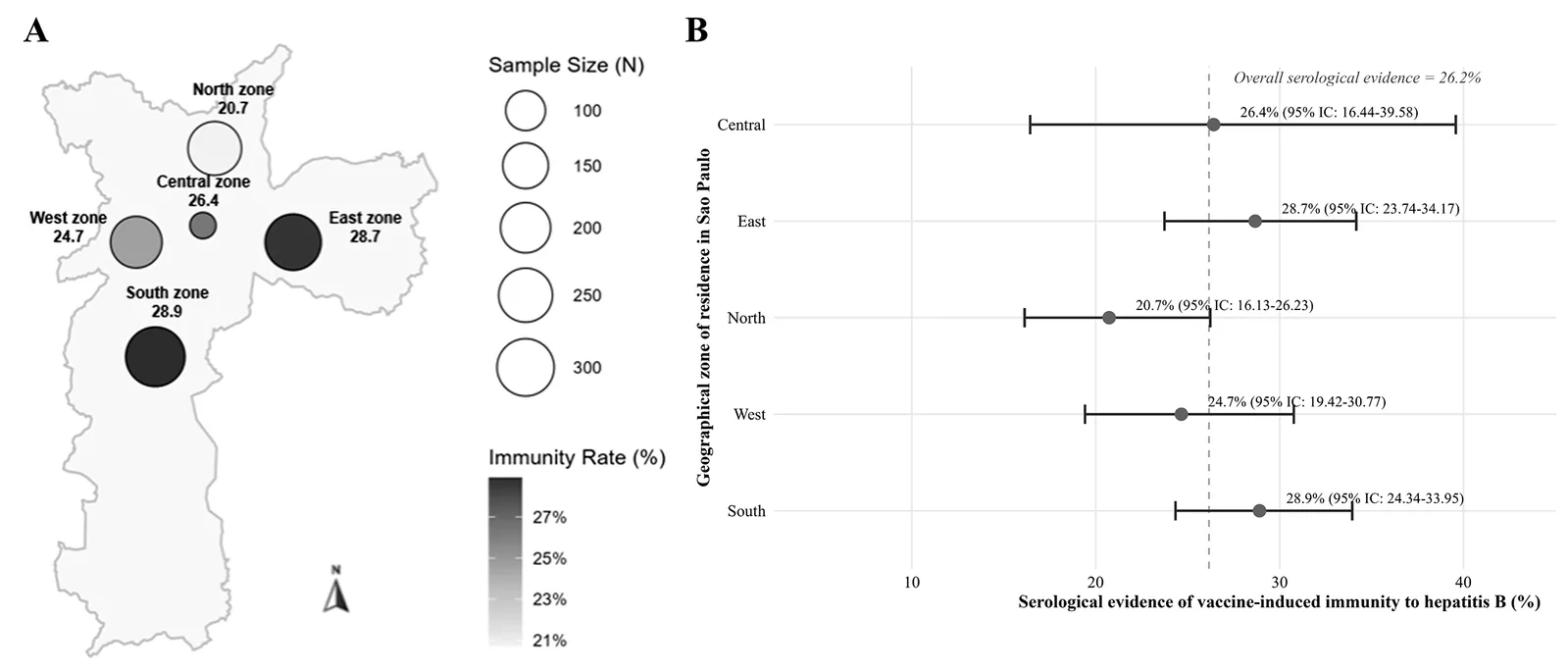
Geographic distribution of serological evidence compatible with vaccine-induced immunity to hepatitis B (anti-HBs) among cancer patients residing in Sao Paulo city, Brazil, 2023: (A) Spatial distribution according to zone of residence, with bubble size proportional to the sample size and color intensity representing the proportion of patients with serological evidence of vaccine-induced immunity; (B) Proportions of patients with serological evidence compatible with vaccine-induced immunity and corresponding 95% confidence intervals (95% CI) by zone of residence. The dashed vertical line represents the overall proportion observed in the study population.

**Table 1:** Associations between zone of residence in Sao Paulo city, epidemiological characteristics, and serological evidence compatible with vaccine-induced immunity to hepatitis B (anti-HBs) among 1,143 cancer patients, 2023.

Characteristic	Isolated anti-HBs		PR (95% CI) crude	p^c^	PR (95% CI) adjusted	p^d^
	Absent n (%)	Present n (%)				
**Age, years**						
18-39	84 (44.7)	104 (55.3)	3.72 (2.79-4.97)	**<0.001**	3.56 (2.66-4.77)	<0.001
40-59	290 (72.0)	113 (28.0)	1.89 (1.42-2.51)	**<0.001**	1.73 (1.30-2.31)	<0.001
≥ 60	470 (85.1)	82 (14.9)	1.00		1.00	
**Sex**						
Female	496 (70.1)	212 (29.9)	1.50 (1.17-1.92)	**0.002**	1.42 (1.10-1.82)	0.007
Male	348 (80.0)	87 (20.0)	1.00		1.00	
**Ethnicity/skin color^a^**						
Non-White	310 (69.4)	137 (30.6)	1.32 (1.05-1.66)	**0.017**	1.15 (0.91-1.44)	0.244
White	532 (76.8)	161 (23.2)	1.00		1.00	
**Primary cancer^b^**						
Hematological	166 (74.8)	56 (25.2)	0.96 (0.72-1.28)	0.777	-	-
Solid	647 (73.7)	231 (26.3)	1.00			
**Zone of residence**						
Central	39 (73.6)	14 (26.4)	0.91 (0.52-1.60)	0.752	0.87 (0.49-1.52)	0.612
East	204 (71.3)	82 (28.7)	0.99 (0.74-1.33)	0.956	0.98 (0.73-1.31)	0.871
North	195 (79.3)	51 (20.7)	0.72 (0.51-1.01)	0.054	0.77 (0.55-1.09)	0.139
West	165 (75.3)	54 (24.7)	0.85 (0.61-1.19)	0.348	0.81 (0.58-1.12)	0.202
South	241 (71.1)	98 (28.9)	1.00		1.00	

^a^3 no data; ^b^43 no data; ^c^Univariable Poisson regression; ^d^Multivariate Poisson regression; Anti-HBs = hepatitis B surface antibody; CI = confidence interval; PR = prevalence ratio.

**Table 2:** Epidemiological characteristics of cancer patients according to zones of Sao Paulo city, 2023.

Zone of residence							
Characteristic	TotalN = 1,143 n (%)	Centraln = 53 n (%)	East n = 286 n (%)	North n = 246 n (%)	West n = 219 n (%)	South n = 339 n (%)	p
**Age, years**							0.184
18-39	188 (16.4)	9 (17.0)	49 (17.1)	33 (13.4)	45 (20.5)	52 (15.3)	
40-59	403 (35.3)	22 (41.5)	100 (35.0)	77 (31.3)	71 (32.4)	133 (39.2)	
≥ 60	552 (48.3)	22 (41.5)	137 (47.9)	136 (55.3)	103 (47.0)	154 (45.4)	
**Sex**							0.504
Female	708 (61.9)	34 (64.2)	177 (61.9)	142 (57.7)	144 (65.8)	211 (62.2)	
Male	435 (38.1)	19 (35.8)	109 (38.1)	104 (42.3)	75 (34.2)	128 (37.8)	
Ethnicity/skin color^a^							0.835
Non-White	448 (39.3)	20 (37.7)	114 (39.9)	88 (36.2)	90 (41.1)	136 (40.1)	
White	692 (60.7)	33 (62.3)	172 (60.1)	155 (63.8)	129 (58.9)	203 (59.9)	
**Primary cancer^b^**							0.340
Hematological	222 (20.2)	11 (21.6)	44 (15.9)	49 (20.3)	47 (21.7)	71 (22.5)	
Solid	878 (79.8)	40 (78.4)	232 (84.1)	192 (79.7)	170 (78.3)	244 (77.5)	
**Isolated anti-HBs**							0.180
Absent	844 (73.8)	39 (73.6)	204 (71.3)	195 (79.3)	165 (75.3)	241 (71.1)	
Present	299 (26.2)	14 (26.4)	82 (28.7)	51 (20.7)	54 (24.7)	98 (28.9)	

^a^3 no data; ^b^43 no data; Pearson’s chi-square test; Anti-HBs = hepatitis B surface antibody.

## DISCUSSION

A previous study reported serological evidence compatible with vaccine-induced immunity to hepatitis B in 22.1% (5,267/23,854) of adult cancer patients evaluated between 2011 and 20235. Another study reported low hepatitis B vaccination coverage among children with hematological malignancies^6^. This study provides updated data, showing serological evidence compatible with vaccine-induced immunity to hepatitis B in 26.2% of cancer patients residing in Sao Paulo city.

When stratified by the city’s five regions, the highest frequency of serological evidence compatible with vaccine-induced immunity to hepatitis B was descriptively observed in the South (28.9%), whereas the lowest was observed in the North (20.7%); however, these regional differences were not statistically significant after multivariable adjustment. The descriptive regional variation may partly reflect demographic distribution, as the South is the city’s most populous region (approximately 2.7 million inhabitants), whereas the Central zone is the least populated^7^ and accounted for the fewest patients in this cohort. Overall, our findings do not support an independent association between place of residence and serological evidence compatible with vaccine-induced immunity in this population. Nevertheless, previous research on measles vaccination has identified Sao Paulo city as a priority area for strengthening vaccination efforts because of relatively low coverage and its potential influence on surrounding municipalities^8^.

Age-related differences were observed, with substantially higher frequency of serological evidence compatible with vaccine-induced immunity to hepatitis B among younger adults (18-39 years) than among individuals aged ≥ 60 years (55.3% versus 14.9%). Similar age-related patterns have been reported in the United States, where hepatitis B vaccination coverage was higher among adults aged 18-29 years (45.5%) than among individuals aged 30-49 years (24.4%) and aged ≥ 50 years (12.8%)^9^. Several mechanisms may contribute to this finding, but they cannot be distinguished in this study. Older adults were less likely to have been vaccinated during childhood because universal hepatitis B vaccination for newborns was introduced in Brazil only in 1998, with expansion to all age groups occurring in 2015. Additionally, immunosenescence may reduce vaccine responsiveness and the persistence of detectable antibodies over time, as both innate and adaptive immune systems undergo progressive functional decline with aging^10^. Furthermore, among cancer patients, a lower frequency of detectable anti-HBs may also reflect waning antibodies, impaired immune responses related to the underlying malignancy or anticancer treatment, or a combination of these factors. Because information on vaccination history, timing of vaccination, and anticancer treatment was unavailable, these mechanisms could not be evaluated separately.

Importantly, even among younger adults, serological evidence compatible with vaccine-induced immunity remained below expected levels. Previous Brazilian studies have similarly reported detectable anti-HBs in only 68.5% of vaccinated medical students^11^ and 38.9% of children aged 1-15 years^12^, despite completion of the three-dose vaccination schedule. Furthermore, childhood hepatitis B vaccination coverage in Brazil was only 55.3% in 2021, remaining below the national target established by the Ministry of Health^13^.

Sex-related differences were also observed. Women showed a higher frequency of serological evidence compatible with vaccine-induced immunity to hepatitis B than men (29.9% versus 20.0%). This finding is consistent with previous reports showing stronger humoral responses to hepatitis B vaccination among women, including higher anti-HBs levels in healthcare workers^14^ and a higher probability of vaccine non-response among men in Brazilian quilombola communities^15^. Biological differences in immune function, including hormonal influences, sex chromosome-related factors, and immune regulation, have been proposed as possible explanations for these observations^16^. However, because this was an observational study without information on vaccination history or post-vaccination antibody kinetics, our findings should be interpreted as demonstrating an association rather than confirming underlying mechanisms.

While no statistically significant regional differences were identified after adjustment, the low proportion of patients with serological evidence compatible with vaccine-induced immunity to hepatitis B observed in this study has important public health implications. Limited access to healthcare services, socioeconomic barriers, housing insecurity, and vaccine hesitancy have previously been associated with disparities in vaccination coverage in Sao Paulo city^17^ and may also contribute to the observed patterns, although these factors were not evaluated in this study. Therefore, targeted strategies to improve hepatitis B vaccination and serological assessment among cancer patients remain warranted, particularly for older adults and men, who showed the lowest frequency of detectable anti-HBs. A recent systematic review showed that educational interventions, reminders, and patient counseling are effective strategies for improving vaccination uptake among patients with cancer^18^. Additionally, HBV screening before chemotherapy remains low worldwide, with a pooled rate of 57%, although screening rates have increased over time^19^. Expanding HBV screening and strengthening vaccination programs may help reduce the risk of HBV reactivation during anticancer treatment^20^.

### Limitations

This study has several limitations. First, given the retrospective design, selection bias cannot be ruled out because information on the anti-HBs testing strategy was unavailable, and inclusion was restricted to patients with available HBV serological results. Second, information on socioeconomic and structural determinants was unavailable. Third, ethnicity/skin color was analyzed as a dichotomous variable (White versus non-White) because of the small number of participants in specific categories, which may have masked differences among individual racial and ethnic groups. Fourth, this was a single-center study conducted at the largest quaternary oncology hospital in Latin America, and the findings may not be generalized to other healthcare settings. Additionally, documented vaccination records were unavailable, and no national system allows comprehensive retrieval of individual hepatitis B vaccination histories. Consequently, the serological profile evaluated in this study should be interpreted as evidence compatible with vaccine-induced immunity rather than as confirmation of vaccination. Finally, information regarding the timing of anti-HBs testing in relation to cancer diagnosis and anticancer treatment was unavailable. Therefore, it was not possible to distinguish whether low or undetectable anti-HBs levels reflected lack of vaccination, waning antibody levels over time, impaired vaccine response, treatment-related immunosuppression, or a combination of these mechanisms. Despite these limitations, the study provides important epidemiological data on serological evidence compatible with vaccine-induced immunity against hepatitis B among cancer patients and identifies demographic groups that may benefit from strengthened vaccination and testing strategies.

## CONCLUSION

Only 26.2% of cancer patients residing in Sao Paulo city presented serological evidence compatible with vaccine-induced immunity to hepatitis B. Although regional differences were not statistically significant after adjustment, older adults and men were less likely to have serological evidence compatible with vaccine-induced immunity. These findings support the need to strengthen HBV screening and vaccination assessment in oncology care.

## Supplementary Material

Supplementary information are available at https://www.scielo.br/10.48331/SCIELODATA.EOBWJF

Supplementary PDF

## Data Availability

The complete anonymized dataset supporting the findings of this study is available from https://doi.org/10.48331/SCIELODATA.EOBWJF.
