# Supplementary material for: Regional distribution of serological evidence of vaccine-induced immunity to hepatitis B among cancer patients in a city in southeastern Brazil
Source: Rev Inst Med Trop Sao Paulo. 2026 Oct 2;68:e66. doi: 10.1590/S1678-9946202668066 (PMC13641768; doi:10.1590/S1678-9946202668066)
Supplement: Supplementary file 1 [file 1678-9946-rimtsp-68-S1678-9946202668066-md1.pdf]

REVISTA DO INSTITUTO DE MEDICINA TROPICAL DE SÃO PAULO

JOURNAL OF THE SÃO PAULO INSTITUTE OF TROPICAL MEDICINE

SUPPLEMENTARY MATERIAL

https://doi.org/10.48331/SCIELODATA.EOBWJF

Regional distribution of serological evidence of vaccine-induced immunity to hepatitis B among cancer patients in a city in southeastern Brazil

Victória Gonçalves de Paula [1], **Marina Rossi de Camargo Pinto** [1], **Gustavo Manoel Ferreira** [1], **Débora Bignotto Rosane Battaglia** [1,2], **Rafael de Oliveira** [1], **Caroline Manchiero** [1,3], **Bianca Peixoto Dantas** [1,3], **Fátima Mitiko Tengan** [1,4], **Edson Abdala** [1,2,3,4], **Mariana Cavalheiro Magri** [1,3]

[1]Universidade de São Paulo, Faculdade de Medicina, Hospital das Clínicas, Laboratório de Investigação Médica em Hepatologia por Vírus (LIM-47), São Paulo, São Paulo, Brazil

[2]Universidade de São Paulo, Faculdade de Medicina, Instituto do Câncer do Estado de São Paulo, São Paulo, São Paulo, Brazil

[3]Universidade de São Paulo, Faculdade de Medicina, Instituto de Medicina Tropical de São Paulo, São Paulo, São Paulo, Brazil

[4]Universidade de São Paulo, Faculdade de Medicina, Departamento de Infectologia e Medicina Tropical, São Paulo, São Paulo, Brazil

Correspondence to: Mariana Cavalheiro Magri
Universidade de São Paulo, Faculdade de Medicina, Instituto de Medicina Tropical de São Paulo, Av. Dr. Enéas de Carvalho Aguiar, 470, Cerqueira Cesar, CEP 05403-000, São Paulo, SP, Brazil
E-mail: mariana.magri@hc.fm.usp.br

Received: 14 May 2026
Accepted: 20 August 2026

Editor: Marcelo Genofre Vallada

Supplementary Table S1 – Cancer patients' characteristics (n = 1,143).

Characteristic	Total
**Age, years**	
Mean ± SD	57.4 ± 16.42
**Sex, n (%)**	
Female	708 (61.9)
Male	435 (38.1)
**Ethnicity/skin color, n (%)**	
Black	104 (9.1)
Indigenous	1 (0.1)
Pardo	328 (28.7)
White	693 (60.6)
Asian	14 (1.2)
Missing	3 (0.3)
**Cancer type, n (%)**	
Hematological	222 (19.4)
Solid	878 (76.8)
Missing	43 (3.8)
**Zone of residence**	
Central	53 (4.6)
East	286 (25.0)
North	246 (21.5)
West	219 (19.2)
South	339 (29.7)

SD = standard deviation.

This is an open-access article distributed under the terms of the Creative Commons Attribution License.
